# Trace Element Levels in Packaged Ice Cream and Associated Human Health Risks: A Simulation-Based Analysis

**DOI:** 10.3390/foods14172943

**Published:** 2025-08-24

**Authors:** Cigdem Er Caliskan

**Affiliations:** Department of Field Crops, Faculty of Agriculture, Kırşehir Ahi Evran University, Kırşehir 40100, Turkey; cigdemer86@gmail.com or cigdem.ercaliskan@ahievran.edu.tr; Tel.: +90-5075103186

**Keywords:** dietary exposure, elemental composition, flame atomic absorption spectrometry, food contamination, risk characterization

## Abstract

This study investigates the concentrations of essential and trace elements (Ni, Cu, Fe, Zn, Mn, and Al) in packaged ice cream samples collected from markets in Kırşehir province, located in Central Anatolia, Turkey, aiming to assess potential health risks associated with their consumption. Among the detected trace elements, Al (3.21–16.6 mg/kg) and Fe (2.03–24.0 mg/kg) had the highest concentrations, followed by Zn (0.56–3.00 mg/kg), Ni (0.84–4.84 mg/kg), Cu (1.15–3.46 mg/kg), and Mn (0.18–1.56 mg/kg). To explore the relationships between trace elements and identify possible contamination sources, chemometric approaches including principal component analysis, correlation matrices, and hierarchical cluster analysis (Ward’s method) were applied. Human health risk assessment was conducted by calculating Estimated Daily Intake (EDI), Target Hazard Quotient (THQ), Hazard Index (HI), and Carcinogenic Risk (CR), with uncertainty evaluated through Monte Carlo Simulation (10,000 iterations). HI values above 1 in children and adults indicate that trace element exposure through ice cream consumption may pose a health risk. High Al-THQ and Ni-CR values in children may require stricter monitoring and regulatory measures in case of long-term and regular consumption.

## 1. Introduction

Ice cream is a popular dessert that is enjoyed by people of all ages, especially during the summer months, and it has a large consumer base all over the world [1]. Ice cream contains significant amounts of milk protein, sugar, fat, and water, along with small amounts of additives (e.g., emulsifiers, stabilisers, colorings, sweeteners). Moreover, ingredients including milk, fruit, nuts, and cacao may serve as sources of trace element contamination [2,3,4,5,6,7,8]. Among these, Ni, Cu, Fe, Zn, and Mn are essential trace elements, while Al is primarily considered a contaminant. Although Fe, Zn, Cu, and Mn are required for various biological functions, certain forms of Ni and Al are associated with toxicological effects [9,10]. Prolonged or excessive intake of foods contaminated with such elements has been linked to a wide range of adverse health outcomes, including hepatotoxicity, neurotoxicity, Alzheimer’s disease, behavioural disorders, tissue damage, and carcinogenesis [11,12]. Given their dual nature as both essential nutrients and potential toxicants, regular monitoring of these elements in widely consumed foods is imperative. Within this framework, ice cream represents a particular concern due to its popularity among children and sensitive populations [7,13]. Consequently, the determination of trace element levels and the evaluation of associated health risks in ice cream have emerged as important research priorities [7,14,15,16].

Researchers have used different analytical methods to measure trace element levels. These methods include inductively coupled plasma-optical emission spectroscopy (ICP-OES) [17,18], inductively coupled plasma-mass spectrometry (ICP-MS) [19,20], electrothermal and flame atomic absorption spectroscopy (ETAAS and FAAS) [21,22], neutron activation analysis (NAA) [23], and X-ray fluorescence (XRF) [24]. Among these techniques, FAAS (flame atomic absorption spectroscopy) offers certain advantages [25]. For example, it allows samples to be introduced in a reproducible manner, increasing the accuracy and reliability of measurements [26]. In addition, FAAS instruments are more accessible and affordable, making it the preferred method for many laboratories [27,28]. However, FAAS also has significant limitations. Ultimately, the choice of method depends on the objectives of the analysis and the capabilities of the laboratory [29].

In the literature, various studies on ice cream samples have provided significant insights into their chemical composition and safety [7,14,15,16,17,30,31,32]. For instance, a study conducted in Italy found significant differences in element content between industrial and handmade ice cream [30]. In another study, Dhar et al. (2021) analyzed the concentrations of elements (Al, Zn, Cu, Ni, Fe, Cr, Mn, Pb, and Cd) in packaged ice cream samples in Bangladesh and assessed the potential health risks associated with these elements [16].

In Turkey, previous studies on ice cream have primarily addressed its mineral composition and quality attributes, often in the context of enrichment with functional ingredients or general dairy product characterisation, rather than focusing on dietary exposure and health risk assessment [7,14,17,31]. To date, only limited research has investigated the trace element content of ice cream in relation to dietary exposure in Turkey. The present study analysed packaged ice cream samples obtained from supermarkets in the Central Anatolia region (Kırşehir, Turkey) to determine the concentrations of six trace elements (Ni, Cu, Fe, Zn, Mn, and Al) and to assess their potential health risks. These elements encompass both essential nutrients (Fe, Zn, Mn) and potential contaminants (Ni, Al), with health outcomes largely dependent on concentration and exposure levels. Importantly, this research represents one of the first studies in Turkey to combine chemometric analysis with Monte Carlo Simulation (MCS)-based probabilistic risk assessment in packaged ice cream, thereby providing novel insights into methodological application and public health implications.

## 2. Materials and Methods

### 2.1. Chemicals and Materials

High-purity-concentrated HNO_3_ (65%), H_2_O_2_ (30%), and HClO_4_ (72%) were purchased from Merck and used without further purification. All glassware used was treated with a 10% HNO_3_ solution for at least 24 h and then thoroughly rinsed with deionized water before use. Plastic containers, polypropylene bottles, pipette tips, Pyrex glass digestion tubes, and all samples or standards in contact with reagents were carefully checked for contamination. Ultrapure deionized water with a resistivity of 18.2 MΩ was obtained from a Milli-Q Plus water purification system (Millipore, Bedford, MA, USA). All dilutions were prepared using a 1% HNO_3_(aq) solution prepared with pure water.

### 2.2. Sample Collection and Preparation

**Figure 1:**
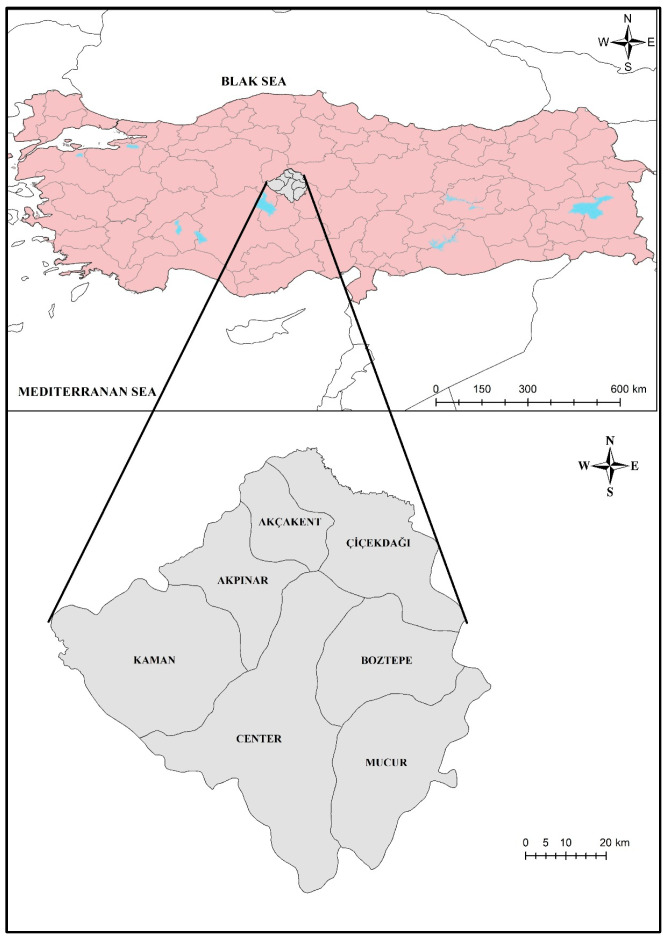
The geographical location of the study area.

This study focuses on ice cream samples collected from Kırşehir province and its districts in the Central Anatolia region of Turkey (Figure 1). To represent the regional distribution and diversity of different brands, a total of 40 packaged ice cream samples from all commercially available brands (*n* = 10) were randomly collected from local supermarkets in Kırşehir during the summer months of 2025. Each sample was recorded with detailed descriptive information, including district of purchase, ice cream type (e.g., cone, stick bar, cup/tub, sandwich, mini, milk-based), flavor (e.g., vanilla, strawberry, chocolate, peanut, almond, pistachio, lemon, berry, goat’s milk), brand (masked), and packaging material (e.g., composite plastic wrapper, composite carton, plastic wrapper, plastic cup). Detailed characteristics of all samples are provided in Supplementary Table S1. In the initial statistical evaluations, only elemental concentrations were considered. However, brand and packaging information were later incorporated into multivariate analyses such as hierarchical clustering (HCA) and principal component analysis (PCA) to explore potential grouping patterns based on trace element concentrations. Three replicates of each ice cream sample were analyzed.

The collected ice cream samples were transported to the laboratory in insulated cold storage boxes containing ice packs to maintain temperature stability during transit and were stored at −20 °C in a deep freezer until analysis. On the day of analysis, plain ice cream samples were unfrozen at room temperature and homogenized using a sterilized stainless-steel spatula. For multi-layered ice creams containing chocolate or similar coatings, the outer coating was carefully removed, and the inner portion was prepared for analysis. Ice cream samples containing fruits or nuts were blended in a commercial kitchen blender to obtain a homogeneous mixture.

From the ice cream samples, aliquots ranging between 0.35 and 0.45 g were weighed, and each sample was subjected to a digestion process in 100 mL temperature- and pressure-resistant PTFE vessels using a microwave digestion system. For digestion, 5 mL of nitric acid (65% HNO_3_, *w*/*w*), 1 mL of perchloric acid (72% HClO_4_, *w*/*w*), and 2 mL of hydrogen peroxide (30% H_2_O_2_, *w*/*w*) were added to each sample. The samples were then digested in the microwave system for 30 min. The resulting solutions were cooled, diluted with 10 mL of ultra-pure water, and filtered using a Whatman No. 541 filter paper (Merck; Darmstadt, Germany). For samples with undissolved residues, the microwave digestion process was repeated.

### 2.3. Trace Element Analysis

Once the ice cream samples were converted into clear solutions, they were analyzed using a High-Resolution Continuum Source Flame Atomic Absorption Spectrometer (HR-CS FAAS, Analytik Jena ContrAA 300, GLE, Berlin, Germany). A blank solution, devoid of ice cream samples, was subjected to the same procedures for comparison. The analysis of the samples was performed in triplicate. The operational parameters of the HR-CS FAAS instrument and the limit of detection (LOD) values for the analyzed trace elements are provided in Table 1. Calibration curves were prepared using trace element stock solutions (Merck) with a concentration of 1000 mg/L, which were diluted to appropriate volumes for analysis.

### 2.4. Data Analyses

To determine the relationships between element concentrations and product composition, statistical analyses such as Pearson Correlation Coefficient (PCC), Principal Component Analysis (PCA), Correlation Coefficient Matrix, and Hierarchical Cluster Analysis (Ward Linkage method) [33] were employed. Health risks associated with trace element exposure through ice cream consumption were assessed using a Monte Carlo Simulation (MCS) with 10,000 iterations at a 95% confidence level [34].

In the MCS model, log-normal distributions were used for trace element concentrations and body weight, a normal distribution for ingestion rate, and a uniform distribution for exposure duration. Other parameters were treated as fixed values. For the health risk assessment, the Hazard Index (HI) and Cancer Risk (CR) values obtained from the simulation were used to calculate the health risk threshold, considering both the mean and the 95th percentile values.

All data analysis and visualization were performed using Python (version 3.13.1), while mapping processes were conducted using QGIS software (version 3.16.4).

### 2.5. Assessment of Health Risks

In this study, a human health risk assessment was conducted for the trace elements Ni, Cu, Fe, Zn, Mn, and Al, considering both adults and children. All six trace elements were detected above the LOD in all samples and were therefore included in the health risk assessment. Within this framework, calculations of Estimated Daily Intake (EDI), Target Hazard Quotient (THQ), HI, and CR were performed. The risk assessment was carried out using the MCS method, which provides a probabilistic approach by accounting for variability and uncertainties in exposure duration.

MCS is a statistical technique that enables the prediction of the range and probabilities of different outcomes by running multiple iterations [35,36]. This method incorporates uncertainties in input parameters, offering a more realistic and comprehensive risk estimation [37,38]. Therefore, MCS serves as an effective tool for better-defining health risks and evaluating the extent of exposure in greater detail [38].

### 2.6. Assessment of Estimated Daily Intake (EDI)

The toxicity level of trace elements with potential health risks depends on their daily intake values [39]. In this study, the EDI values of the trace elements were calculated by considering the trace element concentrations, the daily consumption rate of ice cream products, and the average body weights of adults and children (Equation (1)) [40,41,42].

(1) $$EDI=\frac{MC\times IR}{BW}$$

where

EDI: estimated daily intake mg/kg/day;
MC: trace element concentrations (mg/kg);
IR: average daily ice cream consumption (g/day);
BW: body weight (kg).

Ice cream consumption habits in Turkey were assessed using data from the Association of Packaged Milk and Dairy Products Manufacturers (ASÜD). According to ASÜD, the annual per capita consumption of ice cream in Europe is between 7 and 8 L, while in Turkey, this amount is around 4–5 L [43]. Based on these data, the average daily ice cream consumption in Turkey was calculated to be 10.9–13.7 mL/day. Assuming that the density of ice cream is 0.54 g/mL [44], the consumption amounts were converted from liters to kilograms and the daily consumption was found to be between 5.9 and 7.4 g/day. For dietary exposure calculations, a default BW of 60 kg for adults and 15 kg for children were used, as recommended by FAO/WHO in the absence of country-specific data [45]. The MCS technique was applied with 10.000 iterations, taking into account the distributions of the independent variables (IR, BW, and MC) [46].

The simulation results were visualised using histograms and cumulative probability plots to better understand the consumption distributions and cumulative probabilities. This approach allowed for a more realistic assessment by accounting for individual differences and uncertainties in consumption habits.

### 2.7. Assessment of Non-Carcinogenic Health Risks

To evaluate non-carcinogenic risk, the THQ value was calculated using Equation (2).

(2) $$THQ=\frac{EDI}{RfD}$$

where

THQ: Target Hazard Quotient;
EDI: Estimated Daily Intake (mg/kg/day);
RfD: Reference Dose (mg/kg/day).

RfD is the risk oral reference dose of the element of concern. In this study, the RfD values for Ni, Fe, Mn, Cu, Zn, and Al were 0.02, 0.70, 0.14, 0.04, 0.30, and 0.001 (mg/kg/day), respectively [47,48,49,50,51,52].

The value expressed as Total THQ (TTHQ) or HI (Hazard Index) is calculated from the total THQ values of all trace elements investigated. An HI greater than 1 indicates that there are non-carcinogenic health risks for the consumer [53]. When consuming food, people are exposed not only to one trace element but also to more than one trace element contained in that food. Therefore, the HI was calculated by summing the THQ values of each trace element in the study (Equation (3)). HI < 1 means that there is no concern about health risk, while HI ≥ 1 indicates a potential health problem [53].

(3) $$HI=\sum {THQ}_{i}={THQ}_{1}+{THQ}_{2}+{THQ}_{3}+...+{THQ}_{n}$$

### 2.8. Assessment of Carcinogenic Risks

Carcinogenic risk (CR) is defined as the probability that an individual will develop cancer over a lifetime due to excess exposure to a specific carcinogen [54]. The TCR can be measured using the Cancer Slope Factor (CSF) established by the USEPA [55]. The equation used for TCR assessment (Equation (4)) is provided below [40].

(4) CR=EDI×CSF

where

CR: Carcinogenic Risk;
EDI: estimated daily intake (mg/kg/day);
CSF: Cancer Slope Factor (mg/kg/day).

According to the guidelines set by USEPA, CR values less than 1 × 10^−6^ are considered negligible risk, while values exceeding 1 × 10^−4^ pose a significant hazard to human health [55]. In this context, CR values in the range of 1 × 10^−4^ to 1 × 10^−6^ are considered as manageable or tolerable limits in terms of social acceptability and public health. In this study, CR was assessed only for Ni (1.7) based on available toxicological data [56,57,58,59]. Furthermore, the trace element intake values obtained in this study were compared with the Recommended Dietary Allowance (RDA) and Tolerable Upper Intake Level (UL) values provided in Table 2 for both children and adults. This comparison allowed for a comprehensive evaluation of exposure levels relative to established nutritional guidelines and safety thresholds.

## 3. Results

### 3.1. Trace Element Concentrations in Ice Cream Samples

In this study, the analysis results of the examined trace elements are presented in Table 3. The table includes the minimum (Min.), first quartile (Q1), median, third quartile (Q3), maximum (Max.), mean, and standard deviation (SD) of the element concentrations. The results are expressed in mg/kg, with the detected concentrations of trace element ranging as follows: Ni, 0.84–4.84 mg/kg; Cu, 1.15–3.46 mg/kg; Fe, 2.03–24.0 mg/kg; Zn, 0.56–3.00 mg/kg; Mn, 0.18–1.56 mg/kg; and Al, 3.21–16.6 mg/kg. The accuracy of the method was evaluated using the standard addition method and recovery rates in excess of 95% were achieved, confirming the reliability of the method.

**Figure 2:**
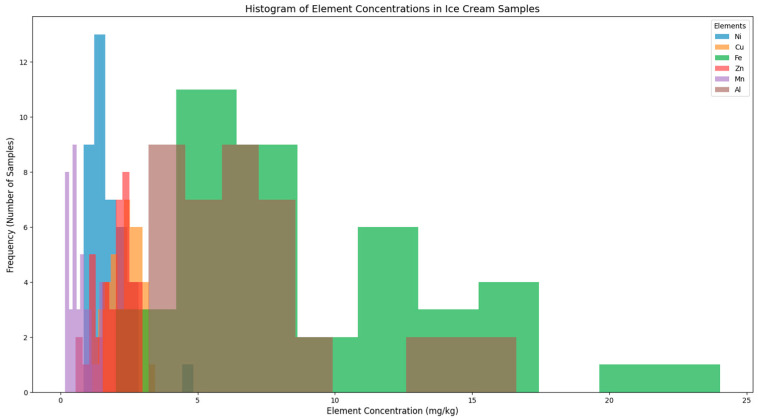
Frequency distribution of trace element concentrations (mg/kg) in ice cream samples. The histograms display the variability of each element across different brands and flavours.

**Figure 3:**
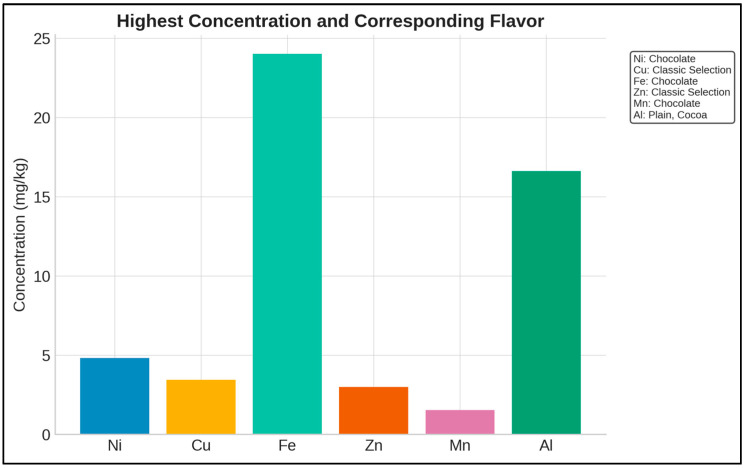
Highest concentrations of trace elements in ice cream samples and corresponding flavors (mg/kg).

The concentration distributions of the trace elements analyzed in the ice cream samples are presented in Figure 2. The trace element concentrations are expressed in units of mg/kg, and the frequency distribution of each element is represented by distinct colors on the graph. This histogram demonstrates that the trace element concentrations in the ice cream samples are generally concentrated at low levels; however, certain trace elements (particularly Fe) reach higher concentrations in some samples. As a result of the analyses carried out on the ice cream samples, Fe and Al stand out as the trace elements with the highest average concentrations. The highest concentrations of each element were detected in specific flavor types (Figure 3). Chocolate samples showed the highest Ni (4.84 mg/kg), Fe (24.03 mg/kg), and Mn (1.55 mg/kg) levels, whereas Classic Selection contained the highest Cu (3.46 mg/kg) and Zn (3.00 mg/kg) concentrations. The highest Al level (16.62 mg/kg) was found in Plain, Cocoa samples.

### 3.2. Principal Component Analysis

**Figure 4:**
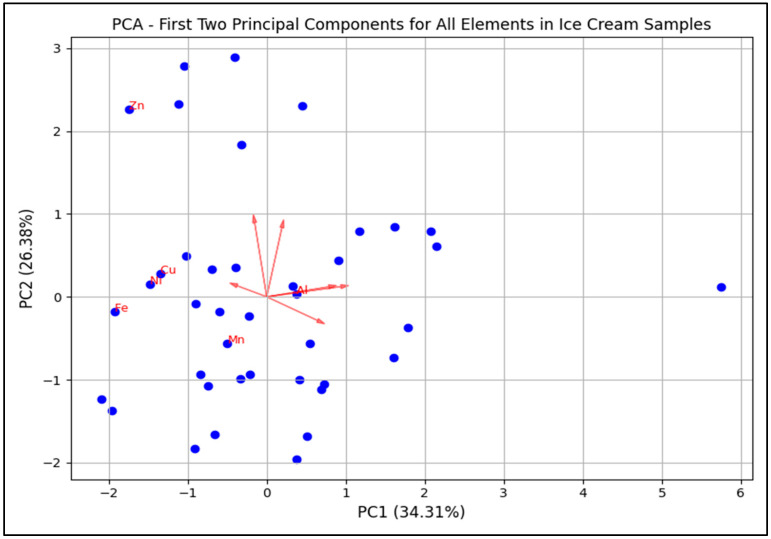
Principal component analysis (PCA) biplot of trace elements in ice cream samples. Each blue dot represents an individual sample, grouped according to elemental composition, while red vectors show the contribution and direction of each element to the principal components (PC1 and PC2).

**Figure 5:**
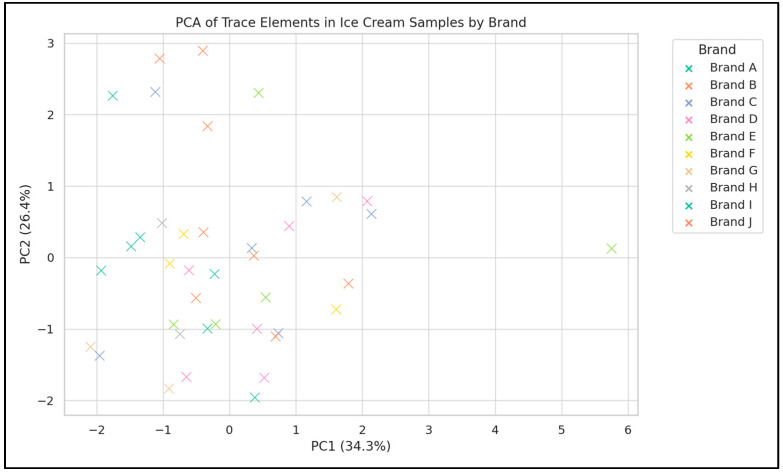
Principal component analysis (PCA) of trace elements in ice cream samples grouped by brand (Brand A–J). Each color represents a different brand. The distribution of samples along PC1 and PC2 illustrates brand-related clustering patterns, while the plot highlights the variation in elemental composition across brands.

In this study, principal component analysis (PCA) was applied to ice cream samples based on their elemental content to uncover brand-related patterns (Figure 4 and Figure 5). The analysis revealed that PC1 and PC2 collectively explained 60.69% of the total variation, summarizing a significant portion of the differences in chemical composition among the ice cream samples (Figure 4). The results indicated that trace elements such as Zn, Al, Fe, and Mn contributed the most to the variation between samples. Specifically, the high loading of Zn on PC2 highlighted its key role in differentiating certain sample groups. The distribution of the samples reflected the similarities and differences in chemical content among specific ice cream groups. The direction and length of the red vectors demonstrated the influence of trace elements on different sample clusters. For instance, samples clustered in the direction of Zn were richer in this element, while trace elements such as Al and Cu exhibited a more homogeneous distribution across other samples (Figure 4). PCA results (Figure 5) showed that PC1 and PC2 explained 34.3% and 26.4% of the total variance, respectively. Samples tended to group by brand, with Brand B generally positioned toward the positive side of PC1 due to higher aluminium concentrations, Brand D clustered in relation to elevated iron levels, and Brand C showing association with higher Ni and Mn contents. These patterns indicate that trace element profiles may be influenced by brand specific raw materials or production processes.

### 3.3. Correlation Matrix Analysis

In this study, Pearson Correlation Analysis was conducted to examine the linear relationships between trace elements (Table 4) [61]. According to the results of the analyses, a significant positive correlation was found between Ni and Mn (r = 0.67, *p* < 0.01), and a moderate correlation between Zn and Al (r = 0.51, *p* < 0.01).

### 3.4. Hierarchical Cluster Analysis: Dendrogram (Ward Linkage)

Hierarchical cluster analysis (HCA) was applied to 40 ice cream samples based on their trace element concentrations to evaluate inter-brand similarities and group samples with comparable elemental profiles. Clustering was conducted using Ward’s linkage method with the Euclidean distance metric [33]. By applying a lower distance threshold (t = 5), five distinct clusters were identified.

**Figure 6:**
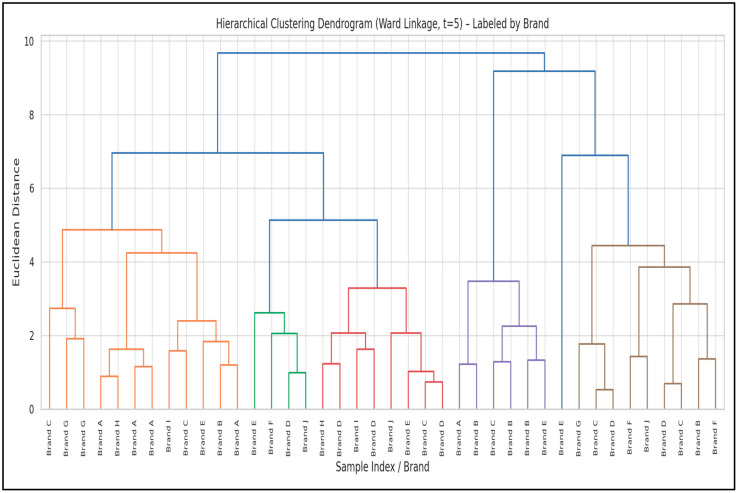
Hierarchical clustering dendrogram of 40 ice cream samples based on trace element concentrations (dendrogram by Ward’s method, labeled by brand).

The resulting dendrogram (Figure 6) depicts the hierarchical organisation of the samples, with the x-axis representing sample indices/brands and the y-axis denoting the Euclidean distance as a measure of dissimilarity. Samples originating from the same brand were predominantly grouped together, reflecting brand-related similarities in elemental composition, whereas notable inter-brand differences were also observed.

Table 5 summarizes the Ward-linkage HCA (t = 5), yielding six clusters (see Figure 6). Cluster sizes (C1–C6) and dominant brands were: C1 (n = 12, Brand A), C2 (n = 4, Brand D), C3 (n = 8, Brand D), C4 (n = 6, Brand B), C5 (n = 9, Brand C), and C6 (n = 1, Brand E). Brand-level signatures are evident: C4—Brand B shows the highest Al (14.8 mg/kg) with relatively high Zn (2.73 mg/kg); C2/C3—Brand D are characterized by elevated Fe (~14–15 mg/kg), with C3 also showing higher Cu (2.82 mg/kg); C5—Brand C is enriched in Ni (2.17 mg/kg) and Mn (1.07 mg/kg); C1—Brand A represents a low-to-moderate background profile; and C6 is a single Brand E outlier with extremely high Fe (24.03 mg/kg) alongside high Ni/Mn. Overall, these clusters corroborate the brand-based separations seen in PCA and point to brand-specific raw materials, processing, and/or packaging as drivers of elemental profiles.

### 3.5. Human Health Risk Assessment

In health risk assessment, MCS (10,000 iterations) was applied to more accurately and reliably characterise exposure risks by incorporating uncertainty into the model [34]. By accounting for probability distributions, this approach enhanced the precision of the analysis for adults and children separately and yielded a more comprehensive risk estimate compared to conventional deterministic methods.

The EDI distributions generated through MCS exhibited a wider range of variability, particularly for Ni and Al in the child group, suggesting notable inter-individual differences in exposure to these trace elements (Table 6).

**Figure 7:**
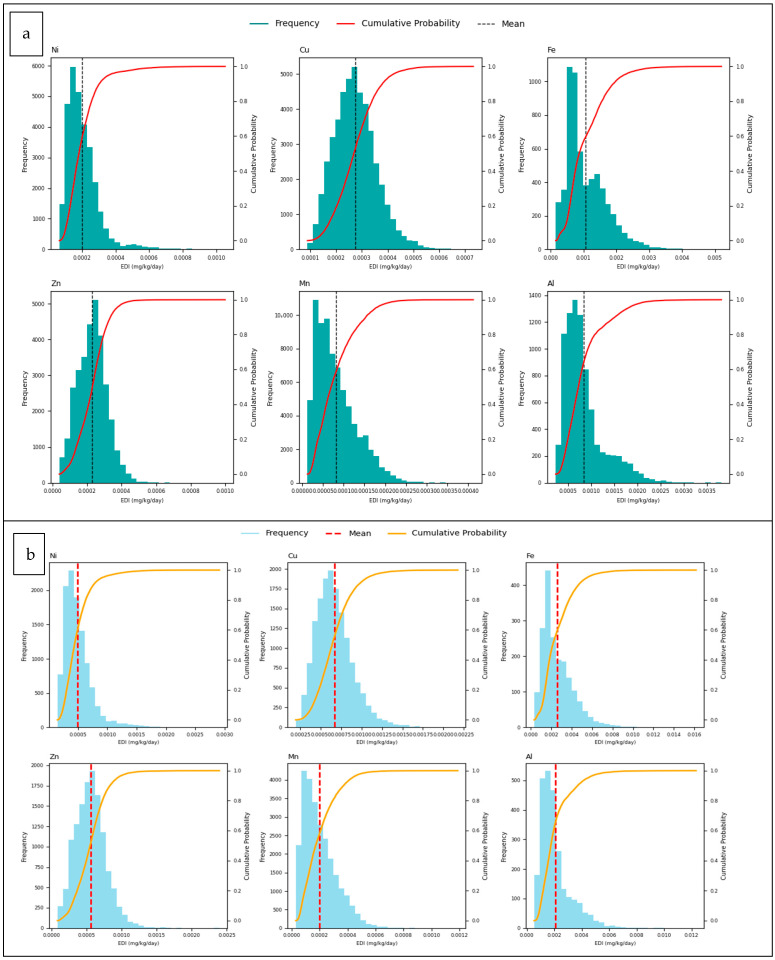
Estimated daily intake (EDI) distribution and cumulative probability of trace element content in ice cream samples by Monte Carlo simulation ((**a**) Adults, (**b**) Children).

Furthermore, Figure 7 presents the EDI distributions and cumulative probability plots obtained through MCS for trace element concentrations in ice cream samples, separately for adults and children.

### 3.6. Non-Carcinogenic Health Risks

The non-carcinogenic health risks of the trace elements studied were evaluated using THQ and HI values (Table 7). The results showed that individual THQ values (Table 7) were generally below 1 for both adults and children, suggesting a low toxicological risk. However, elevated maximum THQ values were observed for Ni (0.14) and Cu (0.075) in children. Notably, the maximum THQ value for Al reached 12.6 in children and 4.28 in adults, far exceeding the safety threshold of 1. Given the established neurotoxic and developmental risks of Al, these findings raise significant public health concerns. HI values supported these results, with maximum values of 12.7 in children and 4.30 in adults. An HI greater than 1 in children underscores their heightened vulnerability due to lower body weight and higher intake rates.

**Figure 8:**
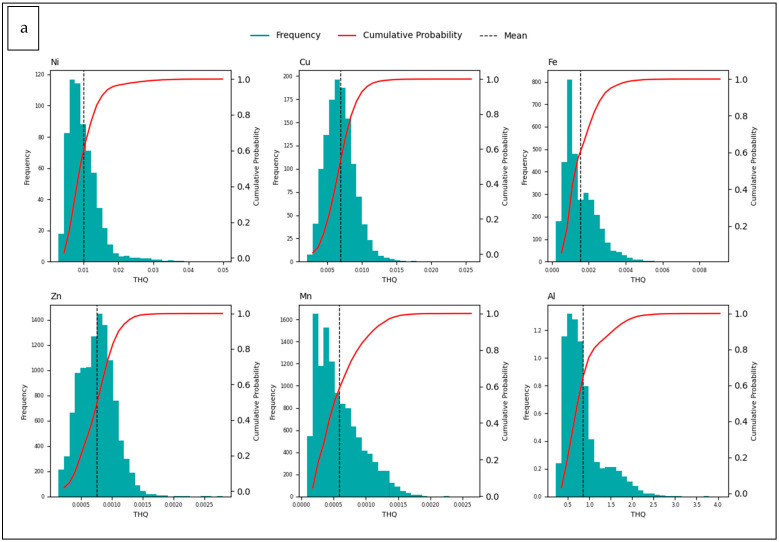

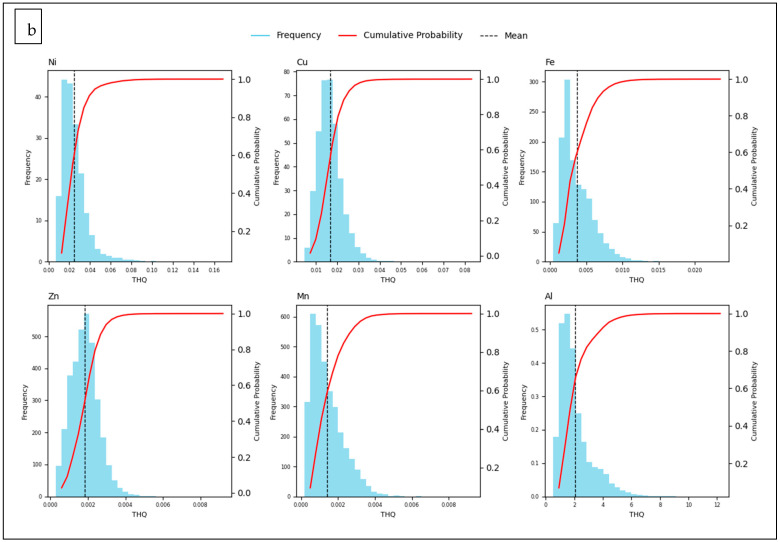
Target hazard quotient (THQ) distribution and cumulative probability for trace element content in ice cream samples using Monte Carlo Simulation ((**a**) Adults, (**b**) Children).

Figure 8 presents the distribution and cumulative probability plots of THQ values obtained from MCS, considering the trace element concentrations in ice cream samples.

### 3.7. Carcinogenic Risk (CR)

**Figure 9:**
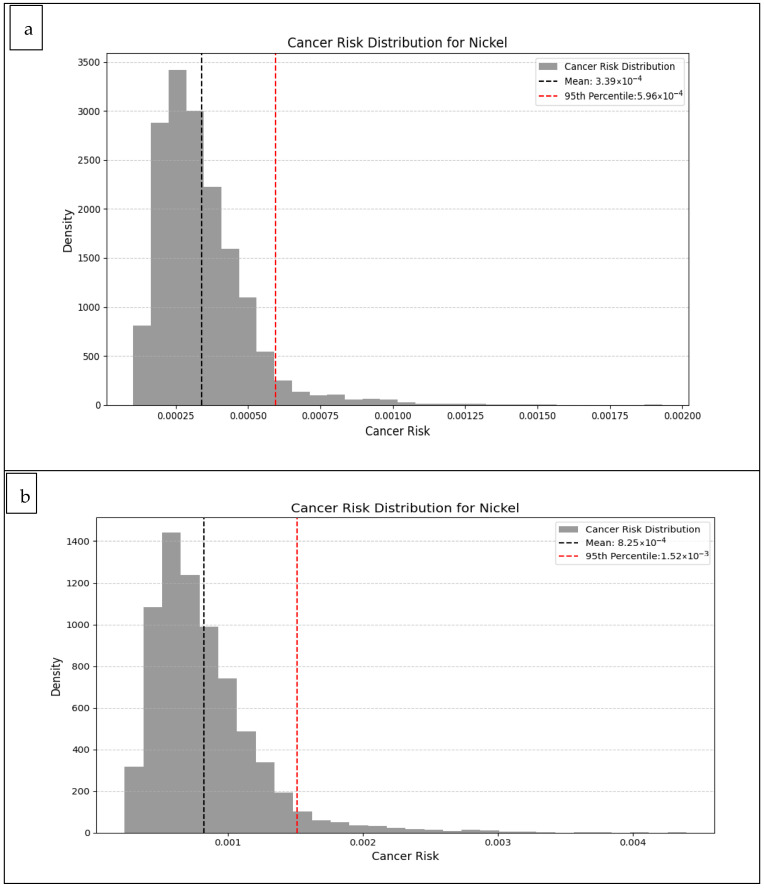
Distribution of Ni cancer risk due to ice cream consumption by Monte Carlo Simulation ((**a**) Adults, (**b**) Children).

In this study, CR values associated with Ni exposure from ice cream samples were estimated using MCS (10,000 iterations) and evaluated separately for adults and children. The distribution for adults (Figure 9a) indicated that most CR values were concentrated at lower levels. The mean CR value was 3.39 × 10^−4^, while the 95th percentile (P95) was 5.96 × 10^−4^ (Figure 9). Although these values exceeded the commonly cited acceptable risk threshold of 1.0 × 10^−4^, they fall within the “moderate risk” range (10^−4^–10^−3^) reported in the literature [16,62]. The CR distribution in the adult group indicates that exposure is at a low–moderate risk level, but that risk approaches the critical threshold value in some individuals.

The distribution graph obtained for the child group (Figure 9b) reveals a more striking risk profile. The average CR value was calculated as 8.25 × 10^−4^, and the 95th percentile value as 1.52 × 10^−3^. These values are significantly higher than those for the adult group, clearly indicating that children are a more vulnerable group to nickel exposure.

## 4. Discussion

This study evaluated the concentrations (Table 3) of six trace elements (Ni, Cu, Fe, Zn, Mn, and Al) detected in packaged ice cream samples, within the framework of health risk assessments. Among these, Ni is an essential micronutrient involved in enzyme function and glucose metabolism, excessive exposure has been linked to hepatotoxicity, nephrotoxicity and neurotoxicity, as well as an increased risk of cancer [63]. According to the World Health Organization (WHO), the tolerable daily intake (TDI) for nickel is 12 μg per kg of body weight. The highest Ni concentration (4.84 mg/kg) was detected in a chocolate-flavored sample packaged in a plastic wrapper. Given that plastic-based packaging is not a significant source of Ni migration, this elevated level is more likely attributable to the cocoa powder content, which is known to accumulate Ni from soil and during processing. Although the measured levels remained within international safety thresholds, the broad range observed between minimum and maximum values suggests considerable variability arising from environmental factors or processing conditions. Similar or lower Ni concentrations in ice cream have been reported in comparative studies [7,16,31]. Nickel contamination is often attributed to stainless steel processing equipment, particularly under prolonged contact or slightly acidic conditions [64]. Additionally, environmental uptake of Ni through feed and water can contribute to its presence in dairy-based ingredients. These findings emphasise the importance of controlling both the sources of raw materials and the conditions of equipment to mitigate Ni-related health risks in ice cream production.

Cu is an essential trace element that plays a vital role in many biological processes, including growth, iron metabolism, and energy production [60]. Cu is a structural component of critical enzymes, including cytochrome c oxidase, ceruloplasmin, and superoxide dismutase. It is also essential for regulating physiological processes, such as neurotransmitter synthesis, connective tissue formation, and immune function [65,66,67]. Cu deficiency can lead to serious health problems, including anemia, osteoporosis, and increased susceptibility to infection [60], while excessive Cu intake can cause liver damage and neurotoxic effects [68]. Therefore, maintaining a balanced Cu intake is of great importance. The RDA for Cu is set at 340–700 µg/day for children and 900 µg/day for adults [60,69]. Both deficiency and excess of Cu can have severe impacts on human health, making balanced intake critically important [70]. The highest Cu concentration occurred in a Classic Selection sample packaged in a composite carton (Table 3). Cu is naturally present in milk and dairy products, but the use of fortified ingredients or minor contributions from internal coatings in composite cartons may also play a role. Literature indicates that Cu concentrations in ice cream can vary significantly depending on the formulation and ingredients used. Products supplemented with additives such as propolis or fruit-based components generally exhibit lower Cu levels, while formulations containing cocoa or nut-derived ingredients tend to have higher Cu concentrations [7,14,16]. Additionally, processing conditions and the use of metallic equipment may further contribute to Cu variability among samples.

Fe is a fundamental component of oxygen-carrying proteins such as hemoglobin and myoglobin, playing a critical role in oxygen transport to tissues [71]. While Fe deficiency can lead to anemia, excessive intake may contribute to conditions such as Alzheimer’s disease, type-2 diabetes, Parkinson’s disease, and various organ damage [72]. The RDA for Fe varies by age and gender: 7–10 mg for children (1–8 years), 11 mg for adolescent males (14–18 years), 15 mg for adolescent females, and 8 mg for adults (51+ years). The tolerable upper intake level is 40–45 mg for children (1–13 years) and 45 mg for adolescents and adults [60].

Fe concentrations displayed considerable variability across the analyzed ice cream samples. The maximum Fe concentration was found in a chocolate-flavored product packaged in a plastic wrapper (Table 3). Plastic is not expected to contribute Fe; therefore, the elevated level likely originates from the cocoa component. Additionally, metallic equipment used during production may contribute to Fe contamination. Similar trends have been reported in previous studies [7,14,16], where ice creams containing ingredients like peanuts, hazelnuts, or cocoa-based components exhibited higher Fe levels compared to vanilla and fruit-based variants [73]. These findings emphasize that raw material composition and processing conditions are key factors affecting Fe concentrations [74]. Given the potential health risks associated with excessive Fe intake, routine monitoring of both ingredient sources and production practices is essential.

Zn is essential for the activity of over 200 enzymes involved in biological processes such as digestion, metabolism, reproduction, and wound healing [75]. Zn deficiency can lead to weakened immune function and cognitive impairments [60], while excessive intake may cause toxicity. Acute Zn poisoning has been associated with gastrointestinal symptoms (abdominal pain, diarrhea, nausea, vomiting) [76]. The RDA for Zn ranges from 3 to 8 mg for children (1–13 years), 9–13 mg for adolescents (14–18 years), and 8–12 mg for adults (19+ years) [60].

The highest Zn concentration was recorded in a Classic Selection sample packaged in a composite carton (Table 3). Zn may derive from milk-based ingredients or stabilizers used in ice cream formulation. Direct migration from the carton packaging is unlikely but cannot be entirely excluded if Zn-containing pigments or coatings are present. Compared to literature data [7,14,16], the Zn levels observed in this study were generally lower, indicating minimal contamination risks. The level of Zn in milk can increase naturally when animals ingest it through their feed or water. However, Zn is a normal component of milk, and trace amounts are expected [77]. Above-normal Zn levels may indicate the presence of equipment contamination or environmental factors. The Zn concentrations observed in this study were consistent with the safe daily intake limit (0.33 mg/day) [60].

Mn is an essential trace element naturally present in many foods and can also be consumed as a dietary supplement [78]. It is involved in the assembly of metalloenzymes such as manganese superoxide dismutase (MnSOD), which is responsible for enzyme activation and scavenging of reactive oxygen species (ROS) during mitochondrial oxidative stress [79]. However, excessive exposure can lead to toxic effects on the central nervous, cardiovascular, respiratory, and reproductive systems [80], while deficiency may disrupt lipid and carbohydrate metabolism, resulting in abnormal glucose tolerance [81]. The highest Mn level (1.55 mg/kg) was shared by two samples—one chocolate-flavored and another honey-almond-vanilla-flavored—both packaged in plastic wrappers. However, the variation observed in manganese levels between different ice cream samples is largely due to differences in recipe composition. Specifically, plant-based ingredients such as cocoa products, nuts, and certain fruits are known to be high in manganese [82]. The manganese content observed in ice cream samples is predominantly attributed to naturally occurring sources, rather than contamination arising from the production process. One of the main contributors is cow’s milk, which naturally contains manganese depending on the mineral composition of the animal’s diet and the geochemical characteristics of the local environment. Soil conditions, such as acidity, play a critical role in the uptake of manganese by forage crops, thereby influencing the elemental profile of milk obtained from grazing livestock [82]. In the literature, Mn concentrations were reported as 0.11–1.03 mg/kg in a study conducted in Bangladesh [16], 0.07–0.15 mg/kg in propolis-supplemented ice creams [7], and 0.20–0.30 mg/kg in another study [14]. Considering the effects of Mn on human health, the RDA for adults is set at 2.3 mg/day, while the tolerable upper intake level (UL) is 11 mg/day [69]. In our study, the consumption of 100 g of ice cream provides approximately 0.072 mg of Mn, corresponding to 3.1% of the RDA. Given that Mn intake primarily occurs through the overall diet, regular monitoring of total intake is essential. However, the Mn levels in our study were found to be within tolerable limits, concluding that ice cream is a safe food product in terms of Mn content.

Al exposure has raised safety concerns due to epidemiological data linking it to Alzheimer’s disease and its adverse neurological, skeletal, hematopoietic, and immunological effects [83]. Children are at greater risk due to their lower body weight and higher surface-to-mass ratio [84]. The intake limit for Al is set at 2 mg/kg of body weight for adults and 1 mg/kg for children [85]. The maximum daily intake is 40 mg for adults and 5–10 mg for children [86,87]. The highest Al concentration (16.62 mg/kg) was detected in a Plain, Cocoa sample packaged in a plastic wrapper, with the second highest (16.44 mg/kg) in a Double-flavored product packaged in a plastic cup. In both cases, the elevated Al may be linked to migration from aluminum-containing layers or additives in the packaging materials, particularly when in contact with dairy matrices. Manufacturing equipment could also contribute to Al contamination. These findings are consistent with previous studies reporting that Al migration is exacerbated under certain storage conditions, such as heat and acidity [7,14,16,85,88]. To minimize potential health risks, it is crucial to implement strict monitoring of packaging material safety and production controls.

These variations may be associated with differences in the source of raw materials, environmental factors (such as soil, water, and air pollution), and production processes. The heterogeneous distribution of element concentrations (Figure 2) suggests that, while the ice cream samples are generally within safe levels, certain exceptional cases warrant more detailed investigation. From a food safety perspective, the potential health risks associated with high concentrations must be considered. Therefore, it is crucial to investigate the sources of samples showing elevated values and the factors contributing to these occurrences. Future studies should explore the origins of these variations in greater detail.

The findings of this study (Figure 3) indicate that the elemental profiles in ice cream samples may be associated with production processes, raw materials used, and environmental factors. The brand-based PCA (Figure 4) revealed variations particularly in Al and Zn that may still be influenced by differences in packaging materials or additive usage. Further supporting this, the PCA score plot (Figure 5), color-coded by brand, showed that ice cream samples tended to cluster according to their respective brands.

These brand-based groupings indicate that trace element profiles reflect brand-specific characteristics and can serve as discriminative markers in assessing product origin, quality consistency, and potential exposure risks. Future studies could provide valuable contributions to food safety and quality control by investigating the causes of these variations and their potential health implications in greater detail.

The significant positive correlation (Table 4) observed between Ni and Mn suggests that these trace elements may originate from similar environmental sources (e.g., raw materials used or water used in production) or agricultural practices (e.g., fertilizer use or soil structure). Dhar et al. (2021) reported that Ni and Mn showed similar concentration profiles in ice cream samples from Bangladesh, possibly due to raw material or waterborne contamination [16]. The moderate positive correlation between Zn and Al suggests a common source, such as additives or packaging materials used in ice cream production. On the other hand, negative correlations such as Ni–Cu (r = −0.26) and Fe–Al (r = −0.32) indicate that these trace elements come from different sources or are influenced by distinct factors in the production process [89,90]. For example, Cu may originate from equipment or production conditions, while Al may come from packaging materials [85]. Hashemi et al. (2017) reported that Cu in dairy products often originates from production equipment, while Al is transferred from packaging to the system [91]. This reveals the risks at different production stages. The low correlations of Zn and Cu with other trace elements support the hypothesis that these trace elements probably originate from specific ingredients or independent processes. For example, Conficoni et al. (2017) reported that Cu originated from production equipment and Zn from additives in industrial ice creams [30].

Dendrogram analysis (Figure 6) identified six distinct clusters, generally reflecting similarities in elemental profiles between samples of the same or similar brands. This structure suggests that differences in elemental composition may be influenced by factors such as brand-specific production lines, raw material sources, or packaging types.

Similar approaches have been applied in previous studies on dairy and processed foods. For example, Hashemi et al. (2017) clustered dairy products based on element content, finding that samples with similar production conditions grouped together [91]. Similarly, Dhar et al. (2021) reported that in ice cream samples in Bangladesh, some samples were grouped in the same clusters due to common sources of contamination (e.g., water, additives) and that these clusters were instructive in risk assessments [16]. In the present study, Brand B samples formed a distinct cluster characterized by high Al concentrations, possibly reflecting unique packaging materials or processing methods (Table 5). Brand C clustered separately due to elevated levels of Ni and Mn, which may be linked to specific ingredient sourcing or equipment (Table 5). Brand D was split into two different clusters, both associated with high iron content, suggesting either product variability or multiple production facilities under the same brand label (Table 5). Interestingly, a single sample from Brand E formed an isolated cluster due to extremely high Fe levels, representing a potential outlier or unique contamination source. The branch lengths in the dendrogram (Figure 6) reflect dissimilarities between brands: shorter branches indicate strong similarity within a brand’s product range, while longer branches reflect pronounced inter-brand variability. This clustering structure provides both a visual and quantitative understanding of brand-specific trace element patterns, offering valuable insights for food safety, quality control, and traceability applications. Samples within the same cluster may benefit from unified monitoring protocols, while samples in separate clusters may require distinct risk assessments.

Consistent with prior studies, HCA proves to be a valuable tool in detecting brand-related variability and guiding risk-based surveillance in processed food products [30,92].

EDI values reflect the exposure of the human body to trace elements through ice cream consumption. In this study, the EDI of each trace elements analysed was compared to the respective Reference Dose (RfD) and Tolerable Daily Intake (TDI) values (Table 6) [93,94]. This study revealed that the average EDI values of the trace elements Ni, Cu, Fe, Zn, Mn, and Al detected in ice cream samples were significantly lower than those obtained in similar studies [16]. Throughout the graphs (Figure 7), it can be seen that the distribution of EDI values is skewed to the right (positively skewed). This means that most EDI values are concentrated at low levels, but there is a tail extending towards higher values. The red dashed line shows the mean EDI value and is generally located on the right-hand side of the distribution, confirming the right skewness. The red (Adult) and yellow (Children) line is the cumulative probability plot. In this study, a wider distribution was observed especially for Ni and Al, indicating that there may be high variation in the exposure levels of these trace elements. This finding is in line with the systematic review by Yan et al. (2022), which emphasised that children are at higher risk than adults, especially for elements such as Al and Ni [92]. On the other hand, the concentration of EDI values for elements such as Fe and Mn at very low levels with low variance suggests that these elements systematically come from limited sources in ice cream production and do not pose a significant public health risk. This is in agreement with the findings reported by Shaala et al. (2022), which showed that Fe and Mn exposure in dried ice cream mixtures was well below acceptable limits [95]. In conclusion, the MCS-based health risk assessment used in this study more reliably revealed uncertainties in trace elements such as Ni and Al, which are of critical importance, especially for children. Compared to similar studies in the literature, it can be concluded that ice cream samples in Turkey have relatively lower risk in terms of EDI levels, but regular monitoring and source-based control strategies should be developed for trace elements with high variation.

The THQ and HI findings (Table 7) obtained are generally consistent with the literature [16,77], but the high values observed in the children’s group warrant closer scrutiny. This situation shows that children are more sensitive to trace elements due to their lower body weight and higher food consumption rates. THQ is a critical parameter in assessing the health risks posed by individual pollutants, providing a more realistic risk analysis by considering variables such as concentration, exposure duration, and body weight [96]. Given the neurotoxic effects and developmental risks of aluminium, the high THQ and HI values detected in children are of serious public health concern. Such high HI values are rarely observed in children in the literature, and these differences can largely be explained by factors such as geographical conditions, environmental pollution levels, raw material quality, and dietary habits. Additionally, the variability in RfD values for Al used in different studies [48] is one of the main reasons for the differences observed in the calculated THQ and HI results.

Although international benchmarks specifically for ice cream do not exist, the European Food Safety Authority (EFSA) has established a Tolerable Weekly Intake (TWI) for aluminium of 1 mg/kg body weight per week [97], while the Joint FAO/WHO Expert Committee on Food Additives (JECFA) suggests a Provisional TWI of 2 mg/kg bw/wk [98]. The distributions obtained indicate that while some individual trace element THQ values (Figure 8) remain below the safe limit of 1, others, particularly Al in children, exceed this threshold. Separate assessments for children and adults show that cumulative exposure (HI) exceeds 1 in both groups, indicating potential non-carcinogenic health risks, especially in children. Such right-skewed distributions and wide ranges of variation indicate individual exposure differences and potential extreme risk groups. Studies by Feizi et al. (2020) and Yan et al. (2022) also reported that as a result of similar simulations, some elements reached HI >1 for children and this situation requires special monitoring [92,99].

These findings underscore the need for regular monitoring and control of element contamination in production processes, with particular attention to sensitive groups such as children.

In particular, although the majority of CR values (Figure 9) in both age groups are below the acceptable limit, the fact that values in the 95th percentile and above exceed the 1.0 × 10^−4^ threshold indicates the presence of high-risk individuals and the need for careful monitoring of exposure in this group. In the children’s group, CR values extending up to 0.004 indicate that this group is more vulnerable to Ni exposure and is at greater risk, particularly in terms of long-term health effects. Similarly, Hasan & Khanam (2021) emphasised that high CR values in children are generally associated with consumption frequency, regional pollution and inadequate regulatory controls [77].

These findings highlight the need for a careful assessment of cancer risk being associated with exposure to nickel in commonly consumed foods, such as ice cream, particularly for children. In the literature, CR values associated with Ni exposure from milk and dairy products are mostly reported in the range of 10^−4^–10^−3^, but values exceeding the 1.0 × 10^−4^ threshold have been reported, particularly in contaminated areas or high-consumption scenarios [16,62,100]. In this context, the average CR value calculated for the child group in our study is above the literature average and indicates a potential public health risk. They also emphasise the importance of taking regulatory measures in this regard.

## 5. Conclusions

In this study, the trace element contents of ice cream samples were analysed and possible health risks for adults and children were evaluated. As a result of the analyses, Fe and Al trace elements were found to have the highest average concentrations, and this was attributed to variations that may result from the source of raw materials, environmental factors, and production processes. Some of the highest individual concentrations were linked to specific flavors or packaging types: chocolate-based products showed the highest Ni, Fe, and Mn levels (likely from cocoa powder), Classic Selection samples in composite cartons had the highest Cu and Zn (possibly from milk-based ingredients or stabilizers), and a Plain, Cocoa product in a plastic wrapper had the highest Al (suggesting possible packaging-related migration). In PCA, especially the high loading of Zn in PC2, revealed that this trace element plays an important role in distinguishing certain groups of samples. Furthermore, negative correlations were found between Ni and Cu, while a strong positive correlation was observed between Ni and Mn. These results suggest that the trace elements come from different sources or have different relationships with each other.

MCS has enabled more accurate handling of uncertainties and variations in risk assessment and provided the opportunity to analyse health risks for adults and children separately. This method contributed to a more comprehensive and reliable assessment of food safety risks compared to deterministic approaches. The mean HI values exceeded 1 in both adults and children, indicating potential non-carcinogenic health risks from combined trace element exposure. Notably, HI values in children were higher than those in adults, suggesting that this age group may be more vulnerable due to their lower body weight and higher intake per unit of body weight. The fact that the mean CR value of children for Ni is higher than that of adults reveals different exposure risks between age groups. Considering that children are more sensitive to these risks due to their biological sensitivity, especially during the growth and development period, it is critical to minimize these exposures.

The study has several limitations: The sample was taken from a single region and season; the bioavailability of trace elements was not assessed; only a limited number of trace elements were analyzed; and the nutritional composition of the ice cream samples was not examined. In addition, no consumer survey was conducted to assess actual ice cream consumption habits, which could further refine health risk estimations. Future studies should examine in more detail the root causes of variations in trace element concentrations and focus, in particular, on the raw material supply chain, production conditions, and environmental impacts. Furthermore, regular monitoring and analyses are of great importance to ensure compliance with food safety standards. Such studies will contribute to improving quality control processes in the food industry as well as protecting consumer health.

## Figures and Tables

**Table 1:** HR-CS FAAS Instrument parameters and detection limits.

Variables	Ni	Cu	Fe	Zn	Mn	Al
Wavelength, nm	232.00	324.75	248.32	213.85	279.48	396.15
N_2_O-C_2_H_2_ flow rate, L/h	0	0	0	0	0	215
C_2_H_2_-Air flow rate, L/h	55	55	60	60	80	55
Burner height, mm	7	6	5	8	8	7
Evaluation pixels, pm	3	3	3	3	3	3
* LOD (mg/L)	0.0012	0.001	0.001	0.001	0.001	0.022

* 3σ, 11 Repeat.

**Table 2:** Trace element intake values for children and adults (RDA and UL) [60].

Element	Age Group	RDA (µg/day)	UL (mg/day)
Ni	Children		11 *
	Adults		
Cu	Children	340–700	3–5
	Adults	900	10
Fe	Children	7–8	40
	Adults	8	45
Zn	Children	3–8	7–23
	Adults	11	40
Mn	Children	-	2–6
	Adults	2.3	11
Al	Children	-	5–10
	Adults	20	40

RDA: Recommended Dietary Allowances. UL: Tolerable Upper Intake Levels. * µg/kg.

**Table 3:** Descriptive statistics of trace element concentrations in ice cream samples (mg/kg, wet weight basis).

Element	Min.	Q1	Median	Q3	Max.	Mean	SD
Ni	0.84	1.27	1.62	2.14	4.84	1.75	0.72
Cu	1.15	2.03	2.44	2.78	3.46	2.39	0.56
Fe	2.03	5.67	7.17	12.8	24.0	9.30	4.88
Zn	0.56	1.57	2.21	2.43	3.00	2.00	0.62
Mn	0.18	0.42	0.58	0.93	1.56	0.72	0.39
Al	3.21	4.81	6.59	8.19	16.6	7.36	3.59

**Table 4:** Correlation coefficient matrix of trace elements in ice cream samples.

Element	Ni	Cu	Fe	Zn	Mn	Al
Ni	1.00					
Cu	−0.26	1.00				
Fe	0.50 **	−0.01	1.00			
Zn	0.23	0.08	0.05	1.00		
Mn	0.67 **	−0.18	0.18	0.08	1.00	
Al	−0.02	0.07	−0.32 *	0.51 **	−0.01	1.00

Levels of significance: ** *p* < 0.01; * *p* < 0.05.

**Table 5:** Hierarchical cluster analysis of trace elements in ice cream samples (Ward’s Method, t = 5).

Cluster	Ni	Cu	Fe	Zn	Mn	Al	Sample Count	Dominant Brand
1	1.32	2.5	5.41	1.58	0.59	6.37	12	Brand A
2	1.28	2.28	14.07	2.36	0.26	6.96	4	Brand D
3	1.88	2.82	14.56	1.61	0.69	4.27	8	Brand D
4	1.61	2.6	6.01	2.73	0.64	14.8	6	Brand B
5	2.17	1.88	8.27	2.21	1.07	6.64	9	Brand C
6	4.84	1.52	24.03	2.33	1.55	7.27	1	Brand E

**Table 6:** Estimated daily intake (EDI; mg/kg BW day) values for each trace element in ice cream samples.

Element	Min.	Q1	Median	Q3	Max.	Mean	SD	TDI *
For Adults								
Ni	6.20 × 10^−5^	1.37 × 10^−4^	1.80 × 10^−4^	2.38 × 10^−4^	9.56 × 10^−4^	1.99 × 10^−4^	9.00 × 10^−5^	0.012
Cu	8.50 × 10^−5^	2.13 × 10^−4^	2.67 × 10^−4^	3.23 × 10^−4^	8.67 × 10^−4^	2.73 × 10^−4^	8.30 × 10^−5^	0.5
Fe	1.49 × 10^−4^	6.26 × 10^−4^	8.70 × 10^−4^	1.44 × 10^−3^	5.29 × 10^−3^	1.07 × 10^−3^	6.12 × 10^−4^	0.8
Zn	4.10 × 10^−5^	1.67 × 10^−4^	2.32 × 10^−4^	2.84 × 10^−4^	7.73 × 10^−4^	2.29 × 10^−4^	8.30 × 10^−5^	0.3
Mn	1.30 × 10^−5^	4.50 × 10^−5^	7.10 × 10^−5^	1.11 × 10^−4^	3.58 × 10^−4^	8.30 × 10^−5^	4.90 × 10^−5^	0.14
Al	2.19 × 10^−4^	5.32 × 10^−4^	7.25 × 10^−4^	9.98 × 10^−4^	3.76 × 10^−3^	8.50 × 10^−4^	4.55 × 10^−4^	0.29
For Children								
Ni	1.40 × 10^−4^	3.30 × 10^−4^	4.39 × 10^−4^	5.87 × 10^−4^	3.24 × 10^−3^	4.90 × 10^−4^	2.37 × 10^−4^	0.012
Cu	1.81 × 10^−4^	5.12 × 10^−4^	6.42 × 10^−4^	7.89 × 10^−4^	2.56 × 10^−3^	6.69 × 10^−4^	2.25 × 10^−4^	0.5
Fe	3.45 × 10^−4^	1.48 × 10^−3^	2.12 × 10^−3^	3.47 × 10^−3^	1.57 × 10^−2^	2.59 × 10^−3^	1.53 × 10^−3^	0.8
Zn	9.50 × 10^−5^	3.95 × 10^−4^	5.47 × 10^−4^	6.85 × 10^−4^	1.86 × 10^−3^	5.55 × 10^−4^	2.21 × 10^−4^	0.3
Mn	2.90 × 10^−5^	1.04 × 10^−4^	1.68 × 10^−4^	2.67 × 10^−4^	1.55 × 10^−3^	1.99 × 10^−4^	1.24 × 10^−4^	0.14
Al	5.51 × 10^−4^	1.29 × 10^−3^	1.75 × 10^−3^	2.44 × 10^−3^	1.41 × 10^−2^	2.05 × 10^−3^	1.12 × 10^−3^	0.29

* (mg/kg/day).

**Table 7:** Target hazard quotient (THQ) and total hazard index (HI) values for individual trace element in ice cream samples.

Element	Minimum	Q1	Median	Q3	Maximum	Mean	SD
For Adults							
Ni	3.01 × 10^−3^	6.83 × 10^−3^	9.04 × 10^−3^	1.20 × 10^−2^	4.63 × 10^−2^	9.96 × 10^−3^	4.57 × 10^−3^
Cu	2.02 × 10^−3^	5.34 × 10^−3^	6.67 × 10^−3^	8.14 × 10^−3^	2.16 × 10^−2^	6.85 × 10^−3^	2.10 × 10^−3^
Fe	2.23 × 10^−4^	8.95 × 10^−4^	1.24 × 10^−3^	2.07 × 10^−3^	6.06 × 10^−3^	1.53 × 10^−3^	8.72 × 10^−4^
Zn	1.27 × 10^−4^	5.58 × 10^−4^	7.70 × 10^−4^	9.45 × 10^−4^	2.52 × 10^−3^	7.64 × 10^−4^	2.80 × 10^−4^
Mn	9.40 × 10^−5^	3.10 × 10^−4^	4.96 × 10^−4^	7.80 × 10^−4^	2.58 × 10^−3^	5.83 × 10^−4^	3.49 × 10^−4^
Al	2.32 × 10^−1^	5.29 × 10^−1^	7.20 × 10^−1^	9.78 × 10^−1^	4.28 × 10^0^	8.38 × 10^−1^	4.46 × 10^−1^
HI	2.59 × 10^−1^	5.49 × 10^−1^	7.41 × 10^−1^	9.98 × 10^−1^	4.30 × 10^0^	8.58 × 10^−1^	4.46 × 10^−1^
For Children							
Ni	6.81 × 10^−3^	1.65 × 10^−2^	2.18 × 10^−2^	2.90 × 10^−2^	1.43E-01	2.43 × 10^−2^	1.17 × 10^−2^
Cu	4.65 × 10^−3^	1.27 × 10^−2^	1.60 × 10^−2^	1.98 × 10^−2^	7.48 × 10^−2^	1.67 × 10^−2^	5.70 × 10^−3^
Fe	4.53 × 10^−4^	2.11 × 10^−3^	2.99 × 10^−3^	4.87 × 10^−3^	2.39 × 10^−2^	3.68 × 10^−3^	2.20 × 10^−3^
Zn	3.07 × 10^−4^	1.33 × 10^−3^	1.85 × 10^−3^	2.31 × 10^−3^	2.28 × 10^−2^	1.88 × 10^−3^	8.05 × 10^−4^
Mn	2.26 × 10^−4^	7.48 × 10^−4^	1.21 × 10^−3^	1.89 × 10^−3^	7.02 × 10^−3^	1.42 × 10^−3^	8.77 × 10^−4^
Al	5.15 × 10^−4^	1.27 × 10^0^	1.73 × 10^0^	2.41 × 10^0^	1.26 × 10^1^	2.04 × 10^0^	1.13 × 10^0^
HI	5.58 × 10^−4^	1.32 × 10^0^	1.78 × 10^0^	2.46 × 10^0^	1.27 × 10^1^	2.09 × 10^0^	1.13 × 10^0^

## Data Availability

The original contributions presented in this study are included in the article/Supplementary Materials. Further inquiries can be directed to the corresponding author.

## Supplementary Materials

The following supporting information can be downloaded at: https://www.mdpi.com/article/10.3390/foods14172943/s1. Table S1: Characteristics of packaged ice cream samples by district, type, flavour, brand (masked), and packaging material.

## Abbreviations

The following abbreviations are used in this manuscript:

RFD	Reference Dose
RDA	Recommended Dietary Allowances
UL	Tolerable Upper Intake Levels
PCC	Pearson Correlation Coefficient
PCA	Principal Component Analysis
HCA	Hierarchical Cluster Analysis
EDI	Estimated Daily Intake
THQ	Target Hazard Quotient
TTHQ	Total Target Hazard Quotient
HI	Hazard Index
CR	Carcinogenic Risk
SD	Standard Deviation
MCS	Monte Carlo Simulation
EFSA	European Food Safety Authority
TWI	Tolerable Weekly Intake
