# Supplementary material for: Trace Element Levels in Packaged Ice Cream and Associated Human Health Risks: A Simulation-Based Analysis
Source: Foods. 2025 Aug 24;14(17):2943. doi: 10.3390/foods14172943 (PMC12428401; doi:10.3390/foods14172943)
Supplement: Supplementary file 1 [file foods-14-02943-s001.zip › foods-3784727-supplementary.pdf]

Supplementary Table S1: Characteristics of packaged ice cream samples by district, type, flavour, brand (masked), and packaging material.

Sample	District	Ice Cream Type	Flavor	Brand (Masked)	Packaging Material
1	Center	Stick bar	Classic	Brand A	Composite Plastic Wrapper
2	Center	Stick bar	Caramel	Brand A	Composite Plastic Wrapper
3	Center	Cup/Tub	-	Brand A	Plastic Cup
4	Center	Cup/Tub	Peanut, Sour Cherry	Brand A	Plastic Cup
5	Kaman	Milk-based	Almond	Brand B	Composite Carton
6	Kaman	Milk-based	Classic	Brand B	Composite Carton
7	Kaman	Milk-based	Almond	Brand B	Composite Carton
8	Center	Cup/Tub	Double	Brand C	Plastic Cup
9	Mucur	Stick bar	Almond	Brand C	Composite Plastic Wrapper
10	Mucur	Cone	Cocoa	Brand C	Plastic Wrapper
11	Center	Cone	Strawberry	Brand D	Plastic Wrapper
12	Akpınar	Milk-based	Vanilla	Brand D	Composite Carton
13	Akpınar	Cone	Cocoa	Brand D	Plastic Wrapper
14	Center	Milk-based	Plain	Brand E	Composite Carton
15	Center	Cone	Chocolate	Brand E	Plastic Wrapper
16	Çiçekdağı	Cone	Plain	Brand E	Plastic Wrapper
17	Center	Sandwich	Cocoa	Brand F	Plastic Wrapper
18	Center	Cone	Plain	Brand F	Plastic Wrapper
19	Akçakent	Milk-based	Crazy Fruity	Brand F	Composite Carton
20	Center	Cone	Strawberry	Brand G	Plastic Wrapper
21	Kaman	Cone	Strawberry	Brand G	Plastic Wrapper
22	Mucur	Milk-based	-	Brand H	Composite Carton
23	Akpınar	Milk-based	Extra Cream	Brand H	Composite Carton
24	Çiçekdağı	Milk-based	Vanilla	Brand I	Composite Carton
25	Çiçekdağı	Milk-based	Pistachio	Brand I	Composite Carton
26	Boztepe	Cone	Strawberry, Cocoa, Vanilla	Brand J	Plastic Wrapper

27	Center	Cup/Tub	Pistachio, Cream	Brand A	Plastic Cup
28	Center	Cup/Tub	Strawberry, Lemon, Berry	Brand C	Plastic Cup
29	Akçakent	Stick bar	Chocolate	Brand D	Composite Plastic Wrapper
30	Akçakent	Sandwich	Strawberry	Brand D	Plastic Wrapper
31	Kaman	Milk-based	Classic Selection	Brand B	Composite Carton
32	Mucur	Stick bar	Chocolate Carnival	Brand C	Composite Plastic Wrapper
33	Boztepe	Cone	Honey, Almond, Vanilla	Brand G	Plastic Wrapper
34	Center	Milk-based	Chocolate	Brand E	Composite Carton
35	Kaman	Mini	Plain, Cocoa	Brand B	Plastic Wrapper
36	Akçakent	Cone	Plain, Pistachio (Box)	Brand D	Plastic Wrapper
37	Center	Stick bar	Chocolate Delight	Brand J	Composite Plastic Wrapper
38	Mucur	Sandwich	Cocoa, Vanilla	Brand C	Plastic Wrapper
39	Çiçekdağı	Milk-based	Goat's Milk	Brand E	Composite Carton
40	Akpınar	Cone	Plain	Brand J	Composite Plastic Wrapper
